# Supplementary material for: High-Throughput Fluorescent Assay for Inhibitor Screening of Proteases from RNA Viruses
Source: Molecules. 2021 Jun 22;26(13):3792. doi: 10.3390/molecules26133792 (PMC8270262; doi:10.3390/molecules26133792)
Supplement: Supplementary file 1 [file molecules-26-03792-s001.zip › molecules-1235921-supplementary.pdf]

Supporting information

High-throughput fluorescent assay for inhibitor screening of proteases from RNA viruses

Bara Cihlova[1], Andrea Huskova[1], Jiri Böserle, Radim Nencka, Evzen Boura, Jan Silhan*

Institute of Organic Chemistry and Biochemistry of the Czech Academy of Sciences

[1]Co-first authors

*To whom correspondence should be addressed: silhan@uochb.cas.cz;

Supplementary Table S1: The half maximal inhibitory concentration (IC_{50}) values determined for inhibitors of the NS2B-NS3^{pro} and PL^{pro}.

Protease	Inhibitor	IC_{50} (µM)
NS2B-NS3^{pro}	Aprotinin	2.4 ± 0.2
	DTNB	303 ± 54
PL^{pro}	Antabuse	0.1 ± 0.04
	JB-24	1.48 ± 0.39
	2-MP	0.82 ± 0.6
	6-MP	~ 66

Supplementary Table S2: Amino-acid sequences of substrates for the proteolytic reactions of NS2B-NS3^{pro} and PL^{pro}.

PL^{pro} substrate: 6xHisTag-mCherry-TEVsite-ISG15-eGFP

```
MNGSSHHHHHHVSKGEEDNMAIIKEFMRFKVHMEGSVNGHEFEIEGEGEGRPYEGTQTAKLKVTKGG
PLPFAWDILSPQFMYGSKAYVKHPADIPDYLKLSFPEGFKWERVMNFEDGGVVTVTQDSSLQDGEFI
YKVKLRGTNFPSDGPVMQKKTMGWEASSERMYPEDGALKGEIKQRLKLKDGGHYDAEVKTTYKAKKP
VQLPGAYNVNIKLDITSHNEDYTIVEQYERAEGRHSTGGMDELYKSGATENLYFQGAMGWDLTVKML
AGNEFQVSLSSSMSVSELKAQITQKIGVHAFQQRLAVHPSGVALQDRVPLASQGLGPGSTVLLVVDK
CDEPLNILVRNNKGRSSTYEVRLTQTVAHLKQQVSGLEGVQDDLFWLTFEGKPLEDQLPLGEYGLKP
LSTVFMNLRLRGGGTEPGGRSNVSKGEELFTGVVPILVELDGDVNGHKFSVSGEGEGDATYGKLTLK
FICTTGKLPVPWPTLVTTLTYGVQCFSRYPDHMKQHDFFKSAMPEGYVQERTIFFKDDGNYKTRAEV
KFEGDTLVNRIELKGIDFKEDGNILGHKLEYNYNSHNVYIMADKQKNGIKVNFKIRHNIEDGSVQLA
DHYQQNTPIGDGPVLLPDNHYLSTQSALSKDPNEKRDHMVLLEFVTAAGITLGMDELYK*
```

NS2B-NS3^{pro} substrate: 6xHisTag-eGFP-RSSRRSDLVFS-mCherry

```
MNGSSHHHHHHVSKGEELFTGVVPILVELDGDVNGHKFSVSGEGEGDATYGKLTLKFICTTGKLPVP
WPTLVTTLTWGVQCFARYPDHMKQHDFFKSAMPEGYVQERTIFFKDDGNYKTRAEVKFEGDTLVNRI
ELKGIDFKEDGNILGHKLEYNAISDNVYITADKQKNGIKANFKIRHNIEDGSVQLADHYQQNTPIGD
GPVLLPDNHYLSTQSKLSKDPNEKRDHMVLLEFVTAAGITLGMDELYKAMGRSSRRSDLVFSVSKGE
EDNMAIIKEFMRFKVHMEGSVNGHEFEIEGEGEGRPYEGTQTAKLKVTKGGPLPFAWDILSPQFMYG
SKAYVKHPADIPDYLKLSFPEGFKWERVMNFEDGGVVTVTQDSSLQDGEFIYKVKLRGTNFPSDGPV
MQKKTMGWEASSERMYPEDGALKGEIKQRLKLKDGGHYDAEVKTTYKAKKPVQLPGAYNVNIKLDIT
SHNEDYTIVEQYERAEGRHSGGMDELYK*
```

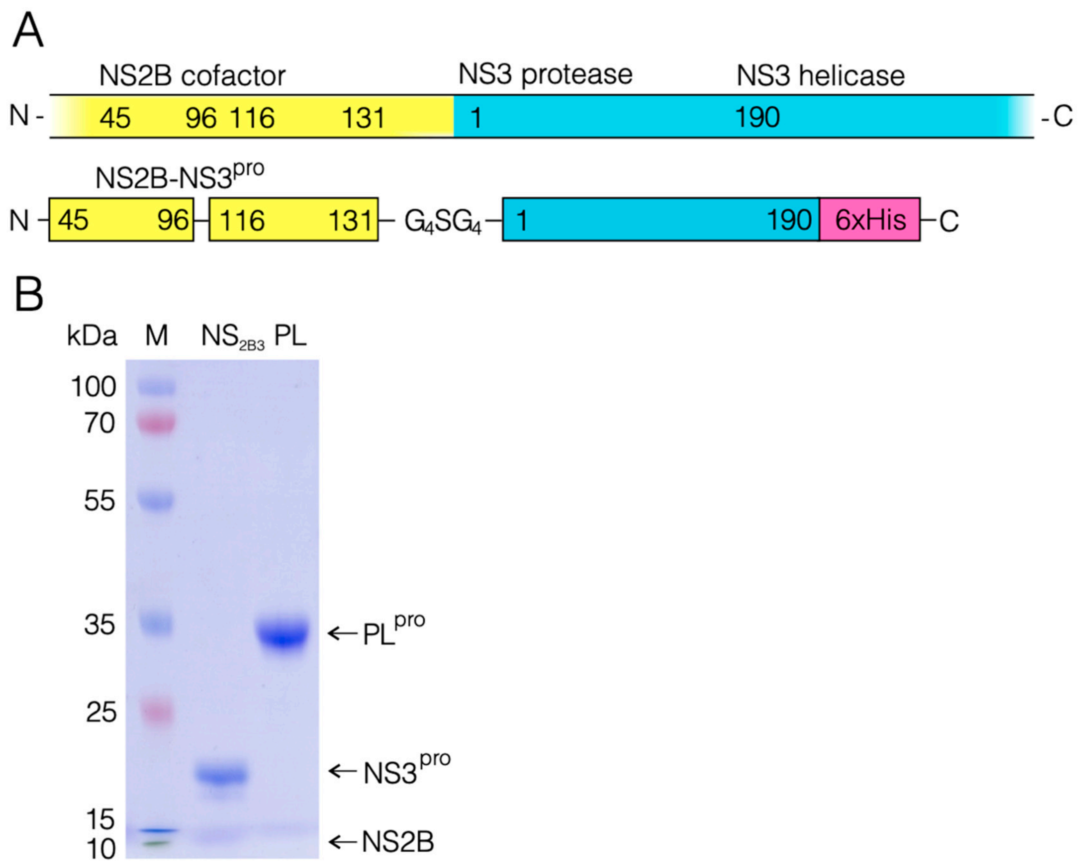

Supplementary Figure S1: Construct of NS2B-NS3^{pro} and validation of molecular masses

(A) To design the NS2B-NS3^{pro}, nonstructural proteins NS2B (residues 45-96) and NS3 (residues 116-131) were connected by a flexible glycine-rich linker. A polyhistidine tag was added to the C terminus to enable the purification. (B) Mass spectrum of the NS2B-NS3^{pro}. (C) SDS-PAGE gel of the NS2B-NS3^{pro} and PL^{pro} where (M) is a marker, (NS$_{2B3}$) is NS2B-NS3^{pro}, (PL) is PL^{pro}.

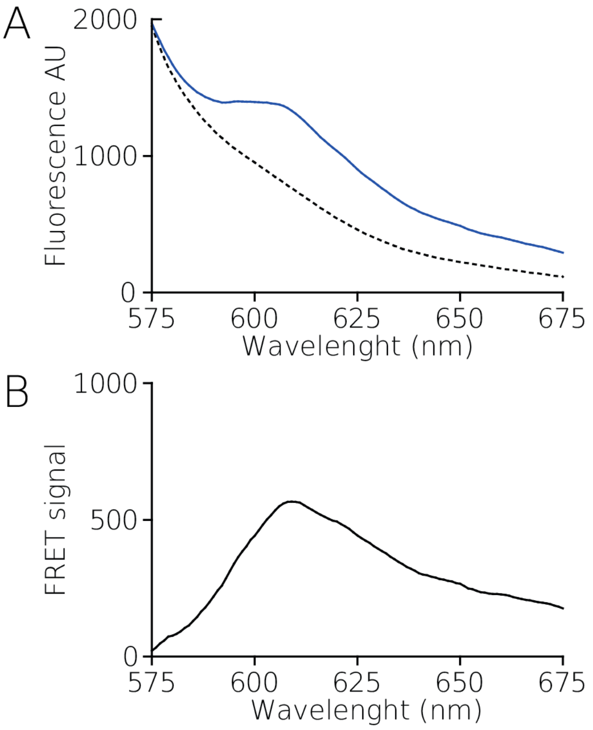

Supplementary Figure S2: Fluorescent emission spectra demonstrating FRET signal loss for PL^{pro} substrate:

(A) fluorescence emission spectra of PL^{pro} substrate (solid-blue line) and product (dashed line) measured before and after the reaction with the enzyme (excitation wavelength was 480 nm), (B) FRET emission, the differential spectrum between the intensity of the substrate and product spectra.

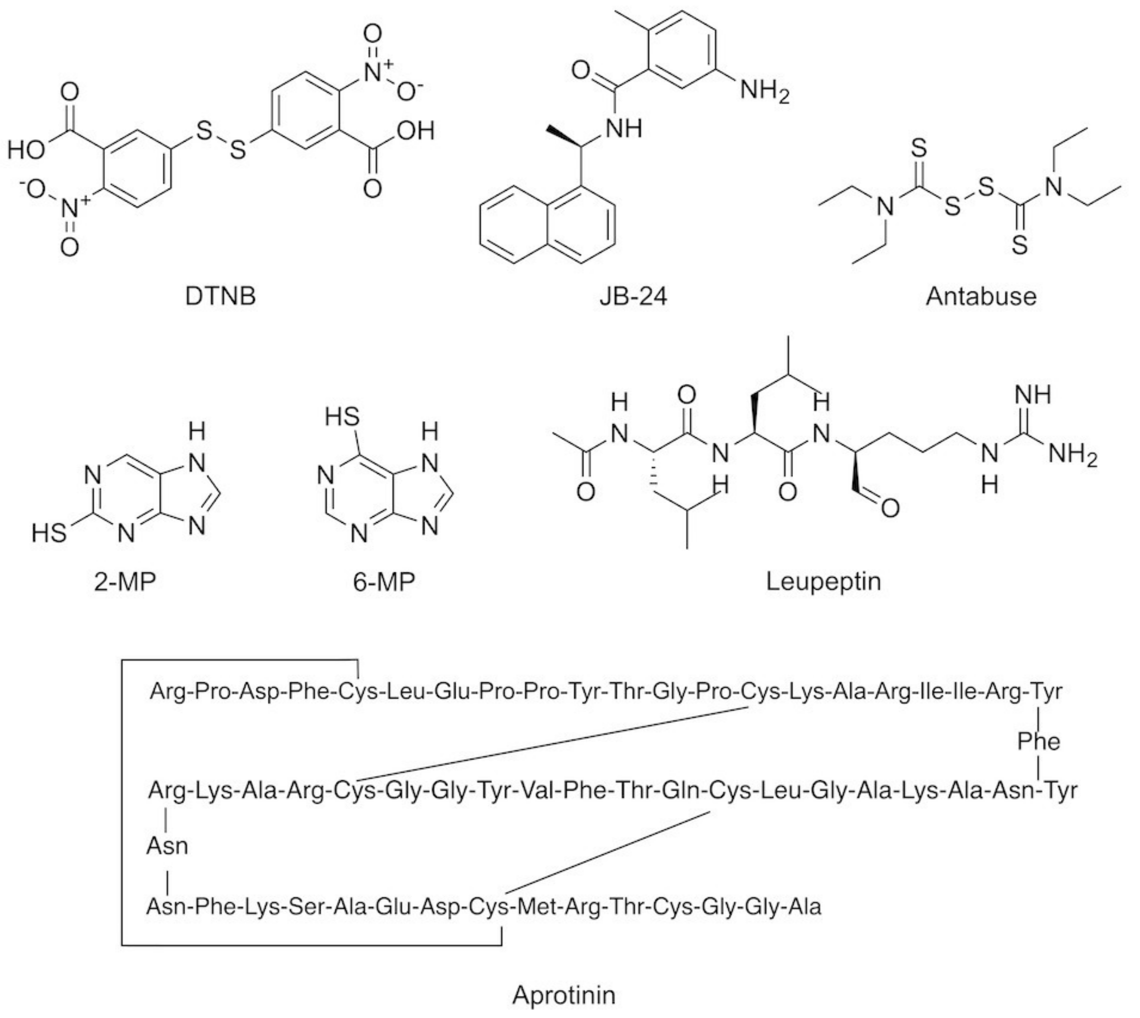

Supplementary Figure S3: Structures of small inhibitor molecules used in this study

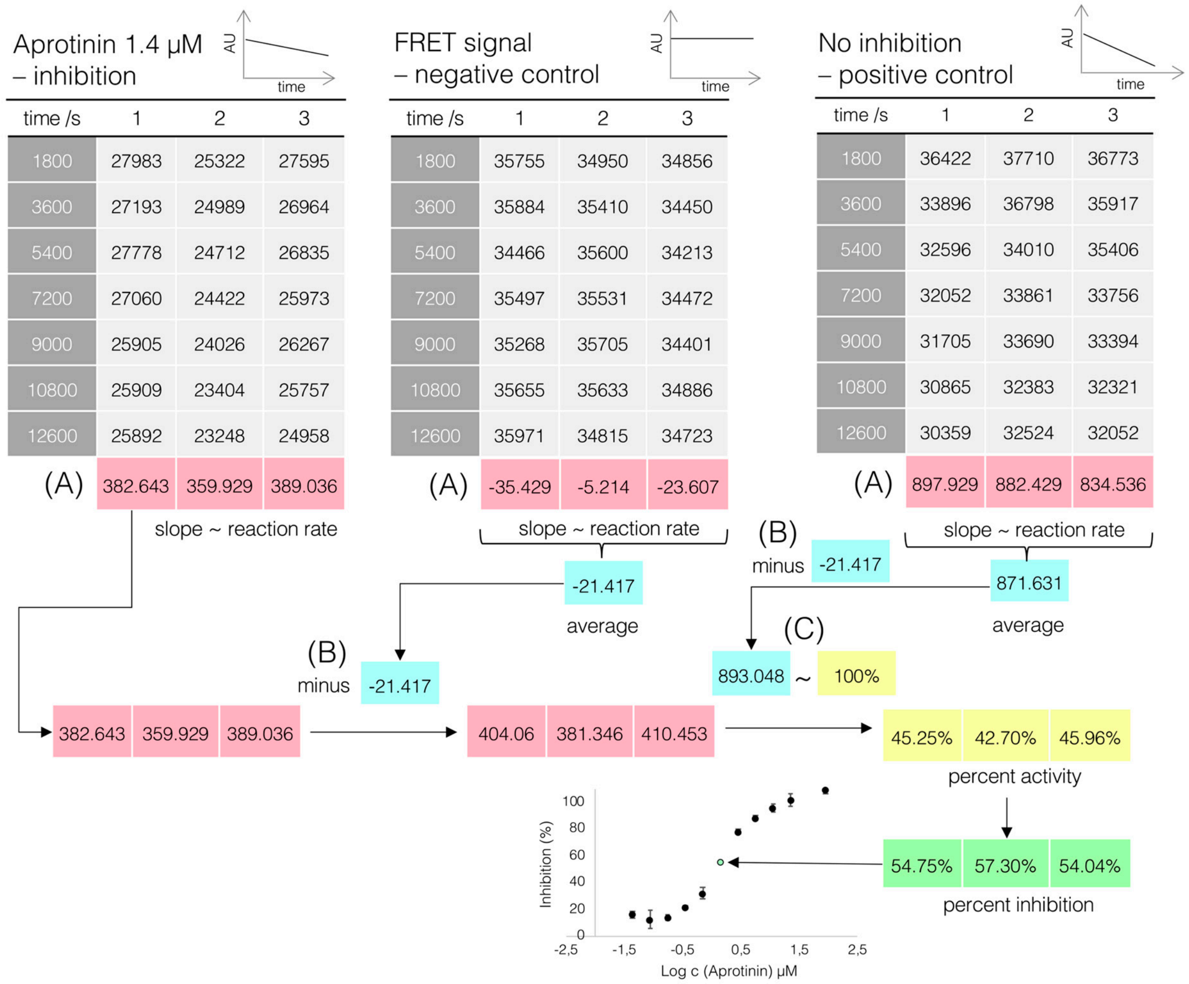

Aprotinin 1.4 µM – inhibition

time /s	1	2	3
1800	27983	25322	27595
3600	27193	24989	26964
5400	27778	24712	26835
7200	27060	24422	25973
9000	25905	24026	26267
10800	25909	23404	25757
12600	25892	23248	24958
(A)	382.643	359.929	389.036

FRET signal – negative control

time /s	1	2	3
1800	35755	34950	34856
3600	35884	35410	34450
5400	34466	35600	34213
7200	35497	35531	34472
9000	35268	35705	34401
10800	35655	35633	34886
12600	35971	34815	34723
(A)	-35.429	-5.214	-23.607

No inhibition – positive control

time /s	1	2	3
1800	36422	37710	36773
3600	33896	36798	35917
5400	32596	34010	35406
7200	32052	33861	33756
9000	31705	33690	33394
10800	30865	32383	32321
12600	30359	32524	32052
(A)	897.929	882.429	834.536

Supplementary Figure S4: Illustrative evaluation of data for a single set of inhibition reactions (NS2B-NS3^{pro} with Aprotinin as an inhibitor).

Tables contain illustrative raw data for one experimental set, each reaction was done in triplicate. (A) The slope of AU versus time was calculated for each well, representing the reaction rate in the corresponding well. (B) Subsequently, the average reaction rate of negative control was subtracted from reaction rates of inhibition reactions and from the average reaction rate of the positive control to correct the data for bleaching and diminishing fluorescence. (C) Corrected rates of inhibition reactions were converted to values of per cent activity and per cent inhibition according to the positive control. The per cent inhibition was plotted against log concentration of the appropriate inhibitor to determine the IC_{50} value of the inhibitor.

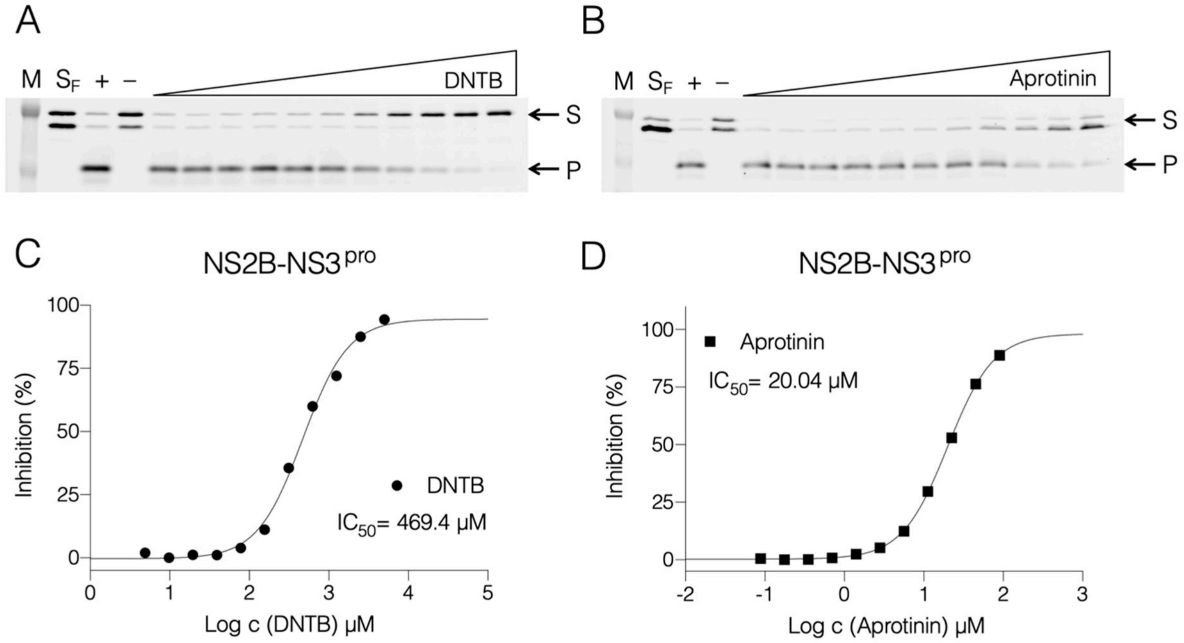

Supplementary Figure S5: Densitometric analysis of TBEV NS2B-NS3^{pro} inhibition by small molecules resolved on SDS-PAGE.

Curves derived from densitometric analysis of the SDS-PAGE gels, (A-B) Fluorescence scans of SDS-PAGE gels depicting proteolytic reaction and inhibition by DNTB (A,C) and Aprotinin (D,B), respectively. Substrate conversions were determined, data were normalized to obtain percentage of inhibition and these were plotted against the Log of concentration of inhibitor and fitted with dose-response curve; (M) is a marker, (SF) is fresh substrate at reaction concentration, (+) is positive control reaction where there was no inhibitor present, (–) is negative control reaction where there was no enzyme present, (S) is the substrate, (P) is the product.

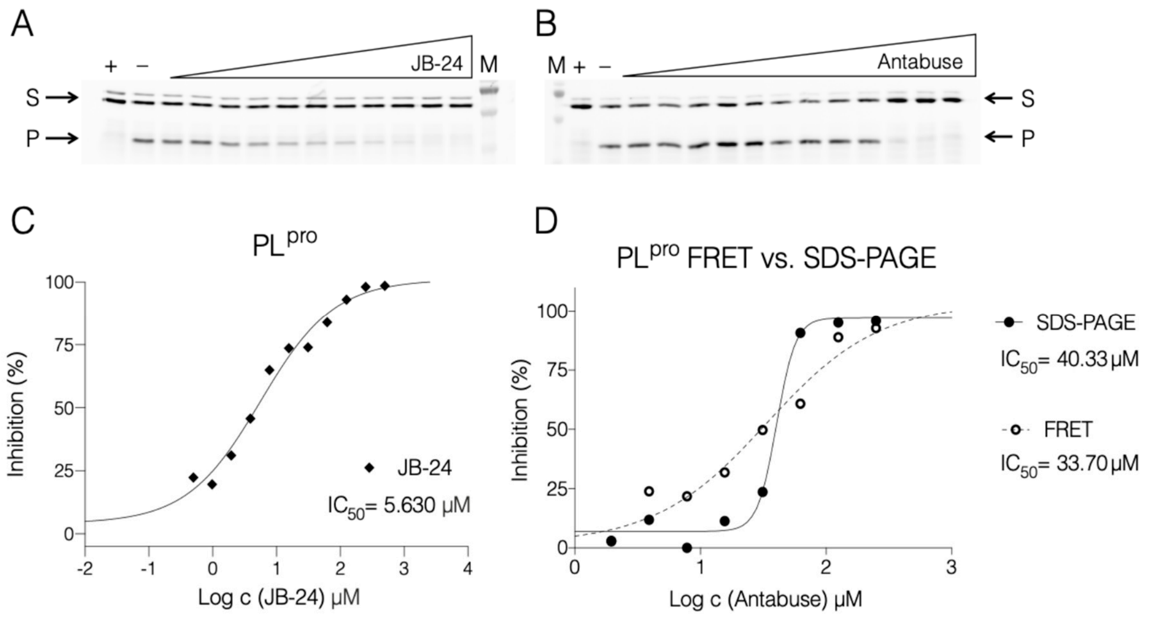

Supplementary Figure S6: Densitometric analysis of SARS-CoV-2 PL^{pro} inhibition by JB-24 resolved on SDS-PAGE and comparative analysis of FRET-based assay and SDS-PAGE assay with inhibitor Antabuse.

Curves derived from densitometric analysis of the SDS-PAGE gels, (A-B) Fluorescence scans of SDS-PAGE gels correspond depicting proteolytic reaction and inhibition by JB-24 (A,C) and Antabuse (D,B), respectively. Evaluated enzymatic data from the gels were plotted against log of concentration of inhibitor and fitted to obtain IC_{50} values; (M) is a marker, (+) is positive control reaction where no inhibitor was present, (–) is negative control reaction where there was no enzyme present, (S) is the substrate, (P) is the product. (D) the example of a single set of reactions with Antabuse titration assayed by both FRET-based assay and SDS-PAGE gel-based inhibition curve. Data were evaluated as above without averaging. The reaction mixture contained residual reducing agent so it cannot be directly compared with data from Figure 3.

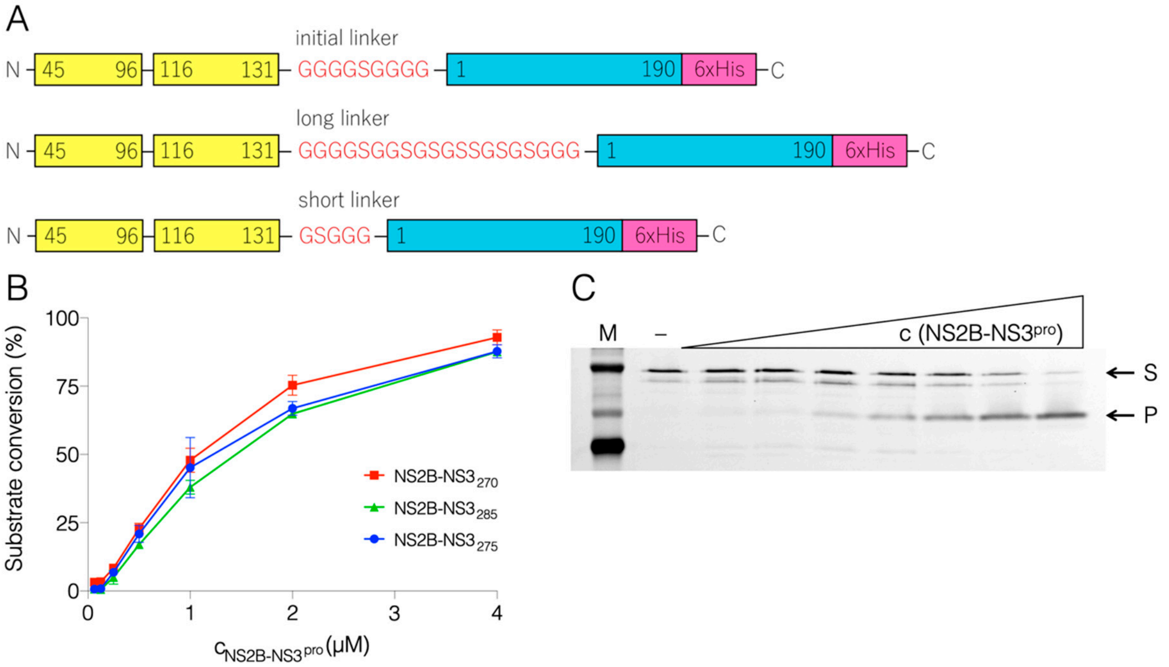

Supplementary Figure S7: Influence of length of the glycine-rich linker between NS2B and NS3

(A) Two additional NS2B-NS3^{pro} constructs with different length of the peptide linker between NS2B and NS3 chains were prepared (longer linker $G_4SG_2SGSGS_2GSGSG_3$ and shorter linker G_1SG_3). These constructs were used to perform gel based assays with NS2B-NS3 FRET substrates, (B) Graphical representation of substrate conversion with increasing concentration of NS2B-NS3^{pro} with short (red), long (green) or original (blue) length of glycine-rich linker between NS2B and NS3, (C) Fluorescent SDS-PAGE gel from one of the titration of NS2B-NS3^{pro} with the original length of the linker, where (M) is a marker, (–) is a negative control without presence of the enzyme, (S) is the substrate and (P) is the product.

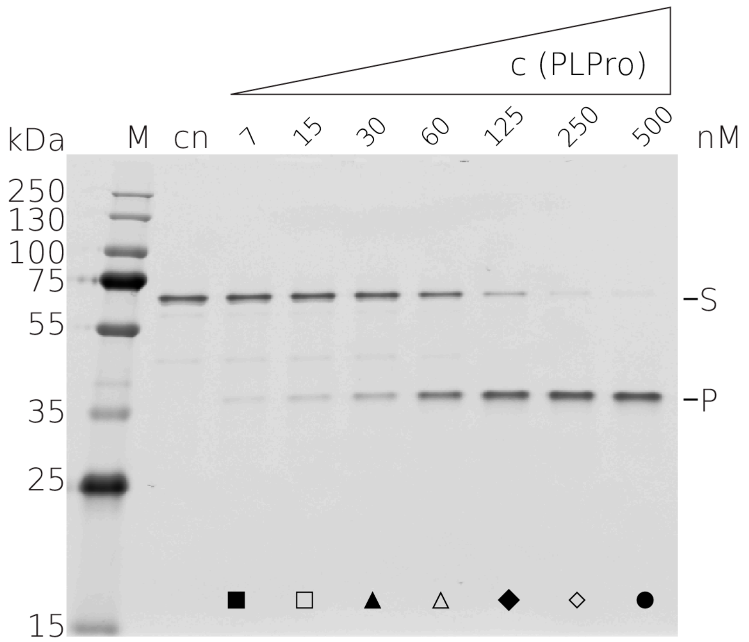

Supplementary Figure S8: Titration of SARS-CoV-2 PL^{pro} protease with fluorescent PL^{pro} substrate resolved on an SDS-PAGE gel.

PL^{pro} substrates were mixed with the increasing amounts of PL^{pro} enzyme, the reaction was quenched after 30 min with SDS sample buffer. The samples were loaded and resolved on 15% SDS-PAGE gel and visualized on a fluorescent scanner (excitation 532 nm laser, emission was collected using LPR 660 nm), where (S) and (P) donate substrate and product bands (this gel represents full-length gel from Figure 1e and the symbols donate symbols used fluorescent measurements plotted in Figure 1d. This experiment was repeated four times.
